# Mechanisms and Design Principles of Proteolysis-Targeting Chimeras and Their Emerging Applications

**DOI:** 10.1021/acsptsci.5c00730

**Published:** 2026-03-16

**Authors:** Nikola Knoll, Nouri Neamati, Aykut Üren

**Affiliations:** † Lombardi Comprehensive Cancer Center, 8368 Georgetown University, Washington, District of Columbia 20007, United States; ‡ Department of Medicinal Chemistry, College of Pharmacy and Rogel Cancer Center, 1259 University of Michigan, Ann Arbor, Michigan 48109, United States

**Keywords:** PROTACs, targeted protein degradation, transcription factors, targeted therapy

## Abstract

The ubiquitin-proteasome system (UPS) comprises an important cellular process that regulates protein homeostasis by selectively degrading misfolded or damaged proteins, as well as those with short half-lives. Proteins are targeted for proteasomal degradation via a multistep cascade of E1-E2-E3 enzymes that catalyze the polyubiquitination of substrate proteins, which can be recognized by the proteasome and degraded into smaller peptides. Targeted protein degradation is a technology that takes advantage of the UPS for selectively channeling proteins of interest (POI) for proteasomal degradation using compounds that facilitate the interaction of an E3 ligase with the POI as a neo-substrate. This review focuses on the main principles of designing proteolysis-targeting chimeras (PROTACs). The advantages and limitations of PROTAC use as a research tool in the laboratory or as novel therapeutics in clinical trials are also discussed. Initially described in 2001, PROTACs have since revolutionized the drug discovery field and comprise a novel therapeutic modality that expands the druggable proteome via a unique mechanism of action. More than 30 PROTACs have entered clinical trials, and early clinical success of PROTACs targeting well-characterized disease drivers inspired the field to move toward targeting proteins that were previously considered “undruggable” with conventional drug design, such as transcription factors. Over the last two decades since its discovery, the PROTAC technology has gained tremendous excitement in both basic research and drug discovery and could soon become a key therapeutic modality. PROTACs can potentially make a significant impact on the survival and quality of life of patients suffering from different diseases.

## Graphical abstract

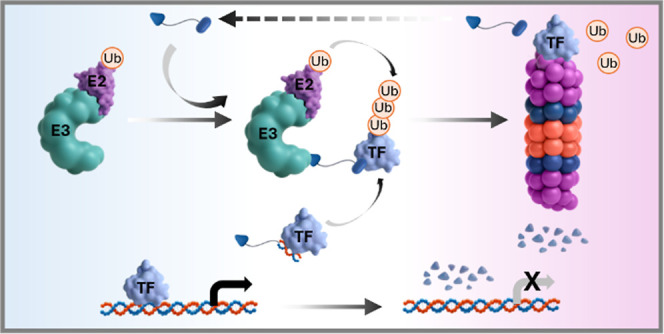

## 1 Introduction

The proteasome is a complex multi-subunit cellular machine that recognizes the polyubiquitination tag on the substrate protein and subsequently degrades it into smaller peptides.^1^ Targeted protein degradation (TPD) is a technology that utilizes the ubiquitin-proteasome system (UPS) for selectively targeting proteins of interest (POI) using compounds that facilitate the interaction of an E3 ligase.^2–5^ Proteolysis-targeting chimeras (PROTACs), first described in 2001, are heterobifunctional small molecules that consist of an E3 ligase ligand linked to a moiety that recruits the POI.^6^ PROTACs have several advantageous properties, including their catalytic-type mechanism that allows them to be active in a sub-stoichiometric manner and, in some cases, do not require high affinity ligands to their targets in a catalytic site, which distinguishes them from classic inhibitors and expands the targetable proteome.^3,5,7^ In this review, the UPS and different TPD strategies will be discussed with a focus on PROTAC design, success in the clinical setting, limitations, and opportunities and an overview of recent reports on PROTACs targeting transcription factors (TFs).

## 2 Ubiquitin–Proteasome System

Proteins are indispensable for cells, as they are involved in virtually every process, including signal transduction, cell movement, structural support, catalysis of biochemical reactions, transport, and cellular defense mechanisms.^8^ Nearly all proteins in the cell are continuously replenished via degrading existing proteins and replacing them with newly synthesized proteins, which is a critical process to maintain cellular homeostasis.^9^ Protein homeostasis comprises complex and interconnected processes that regulate protein concentration, conformation, post-translational modifications, and subcellular localization by controlling protein synthesis, folding, transport, and disposal.^7,10,11^ Importantly, to perform their functions properly, proteins must be folded into the correct three-dimensional structure.^12^ However, misfolding of proteins occurs due to various reasons such as folding mistakes, poor thermodynamic stability of certain conformations, mutations, heat, or oxidative stress, which leaves these proteins in a state of dysfunction and prone to aggregation.^13–15^ Protein aggregation and misfolding can result in severe diseases, including neurodegenerative diseases such as Alzheimer’s, Parkinson’s, and Huntington’s, but have also been linked to aging and cancer, underscoring the importance of cellular strategies to prevent protein aggregation and misfolding.^13,16–19^ The main quality control systems in the cells that keep protein misfolding and aggregation in check are the constant surveillance of proteins by chaperones, as well as protein degradation systems, namely, the UPS and lysosomal proteolysis.^13,14,20–26^ The lysosomes use endocytosis, phagocytosis, or autophagy pathways to degrade long-lived proteins, insoluble protein aggregates, and also larger constructs like entire organelles, macromolecular compounds, and intracellular parasites.^27–32^ The UPS regulates the degradation of approximately 80% of cellular proteins, which highlights its importance in regulating proteostasis, and it is particularly critical for misfolded proteins and proteins with short half-lives, where dysregulation has been shown to be implicated in cancer.^9,33,34^

The proteasome is a crucial component of selective proteolysis via the UPS and comprises multiple subunits that, in concert, enable targeted degradation of numerous proteins in eukaryotic cells which results in irreversible shutdown of the function of that protein.^1^ Given the central role the proteasome plays by regulating protein levels in the cells, it is not surprising that it is crucial in numerous essential cellular pathways such as cell cycle control, major histocompatibility complex I antigen presentation, stress response, apoptosis, and signal transduction.^1,35^ The 26S proteasome in eukaryotes consists of several subunits that make up the 20S core complex^36–45^ and the 19S regulatory particle (Figure 1A).^46–53^ Both entities are important to achieve selectivity. The 19S cap regulates selective substrate recognition and unfolds the substrate protein prior to its transmission into the 20S cylinder containing the proteolytically active sites. Unfolding is a necessary step because the 20S core particle is unable to degrade folded proteins in their native state. Its narrow gate only permits the simultaneous entry of three polypeptide chain stretches, thus requiring unfolding of the substrate protein before degradation.^1^

**Figure 1:**
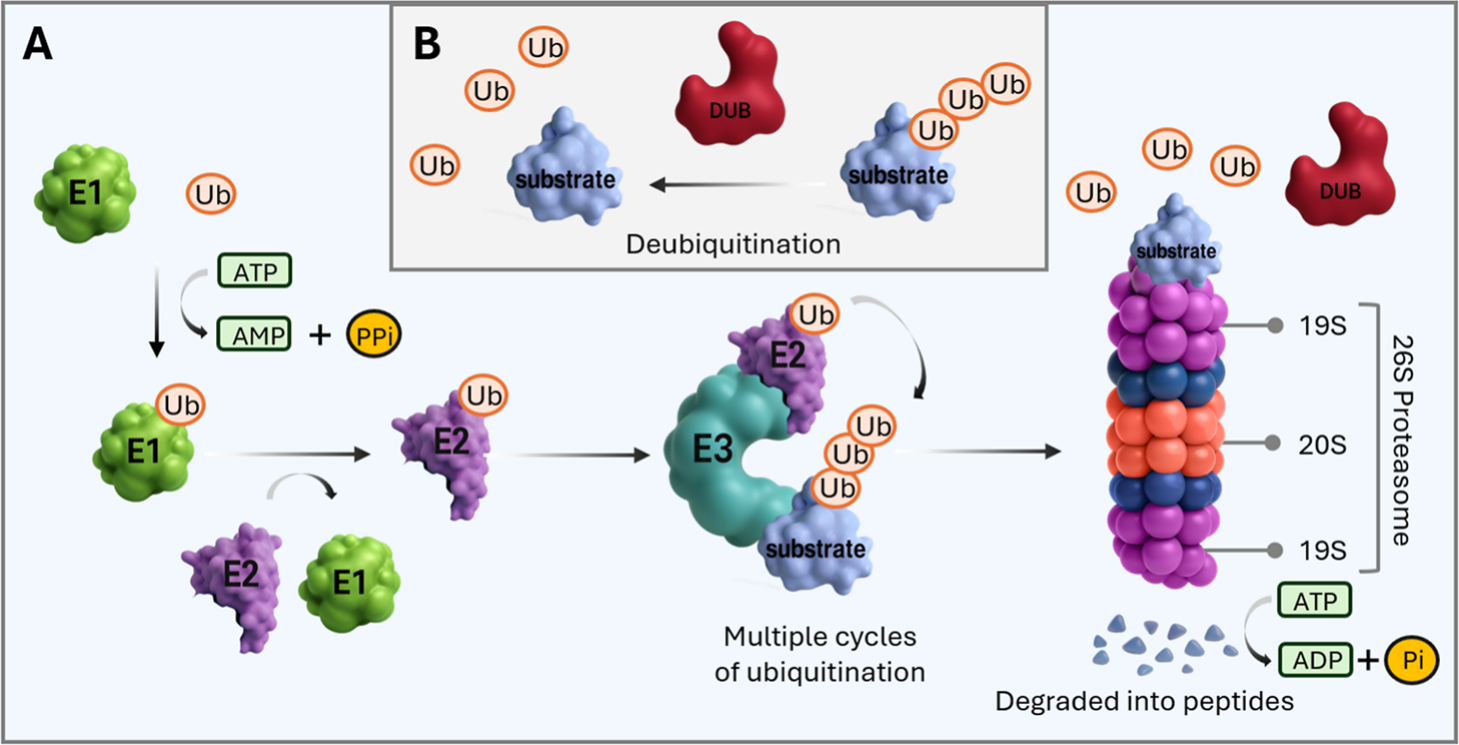
Proteasomal degradation of proteins by the coordinated action of E1-E2-E3 enzymes. (A) Ubiquitin molecules are activated by E1 proteins at the expense of ATP and further transferred to E2 conjugating enzymes. E2 binds to E3 ubiquitin ligases, which recognize substrate proteins and catalyze the ubiquitination of the substrate. Subsequent steps of ubiquitination can destine the substrate for proteasomal degradation, where the polyubiquitin chain is recognized by the 19S regulatory particle. The substrate is then deubiquitinated, unfolded, and threaded through the proteasome into the 20S core particle, consisting of active proteases that degrade the substrate into smaller peptides. (B) Deubiquitination of substrates is mediated by deubiquitinating enzymes.

The 20S complex comprises a stack of four rings, each of which is composed of seven subunits. The two outer and central rings are composed of seven different α subunits and seven different β subunits, respectively. The active sites are located within the central chamber at β subunits of the central rings.^1,35^ The proteolytic activity of the proteasome is described as chymotrypsin-like, trypsin-like, and peptidyl-glutamyl-peptide hydrolyzing activity, cleaving after acidic amino acids, which achieves cleavage of the substrate into small oligopeptide fragments.^42,44,45,54^ Proteins that are destined to be degraded by the proteasome harbor polyubiquitin chains that are recognized by the 19S regulatory cap complex.^1,35^ The yeast 19S particle contains a minimum of 17 different subunits, building the two subcomplexes, the base and lid, which are linked via subunit Rpn10. The base contains a ring of two non-ATPase subunits and six different subunits with ATPase activity of AAA-type that bind to the α rings of the 20S core, while the lid comprises eight different subunits.^55^ In addition to selectively recognizing proteins destined for degradation via binding the polyubiquitin tag, the 19S regulatory particle also mediates unfolding of the substrate, cleaving off the polyubiquitin chain, opening the gates formed by the α subunits on each side of the 20S complex, and, lastly, driving the unfolded substrate into the proteolytic chamber.^1^

Targeting proteins for proteasomal degradation is achieved via tagging the protein with the 76 amino acid polypeptide ubiquitin, which is catalyzed by the coordinated action of three enzymes, E1, E2, and E3, at the expense of ATP (Figure 1). E1 enzymes (ubiquitin-activating enzymes) form a bond with their active site cysteine residue and the ubiquitin’s C-terminal glycine residue, creating an energy-rich thioester bond. The ubiquitin is then transferred to the active site cysteine residue of an E2 enzyme (ubiquitin-conjugating enzyme), from where it can then be linked to a target protein lysine residue, catalyzed by the E3 enzyme (ubiquitin ligase), creating an isopeptide bond. A polyubiquitin chain is formed on the target protein by linking new molecules of ubiquitin to lysine residues, mostly K48, of the existing ubiquitin (Figure 2A,B). Such polyubiquitin chains of at least three ubiquitin moieties linked via lysine 48 residues can be recognized by the proteasome, which degrades the ubiquitin-tagged protein.^1,35,43,56–65^ In addition to K48-linked ubiquitin chains, there are rare cases in which proteasomal degradation was identified via mechanisms beyond polyubiquitin tagging.^66–68^ In line with this, other ubiquitin chain linkages,^69–72^ monoubiquitination,^73–77^ multiple monoubiquitination events,^78,79^ or linkage of ubiquitin on residues other than lysine, such as cysteine, serine, or threonine, were identified to be signals for proteasomal degradation.^80–84^ The covalent attachment of ubiquitin or ubiquitin-like proteins (e.g. NEDD8 and SUMO) to substrate proteins via an isopeptide bond is a reversible post-translational modification regulating critical cellular processes, including cell division, immune responses, and development, and its dysregulation has been observed in diseases such as cancer, neurodegenerative disorders, and muscle atrophy.^85^ Ubiquitin proteins harbor seven key lysine residues, namely, K6, K11, K27, K29, K33, K48, and K63, each of which can form different ubiquitin linkage types (Figure 2C).^86^ In addition to the seven lysine residues, one methionine residue can be used to form a total of eight different polyubiquitin chains, depending on which ubiquitin residue is conjugated.^87^ Ubiquitin linkage via K48 and K63 is well studied and is an important signal for proteasomal degradation as well as intracellular signaling regulating DNA damage repair, cytokine signaling, or autophagic degradation.^88–91^ Other “atypical” ubiquitin linkages have been reported to play roles in DNA damage response (K6),^92^ regulation in the cell cycle, proteasomal degradation, innate immune system, membrane trafficking (K11),^93–95^ protein secretion, DNA damage, mitochondrial damage response (K27),^96,97^ proteasomal degradation, innate immune response, and AMPK related protein kinase regulation (K29),^98–100^ as well as innate immune response and intracellular trafficking (K33).^101,102^ Moreover, three types of ubiquitination modifications on substrates can be distinguished: (1) mono-ubiquitination, where the substrate is ubiquitinated by a single ubiquitin, (2) multi-monoubiquitination, which is characterized by many different single ubiquitin proteins that are attached to different lysine residues on the substrate at the same time, and (3) polyubiquitination, where a single lysine residue on the substrate is labeled with multiple ubiquitin proteins (Figure 2).^9,103,104^

**Figure 2:**
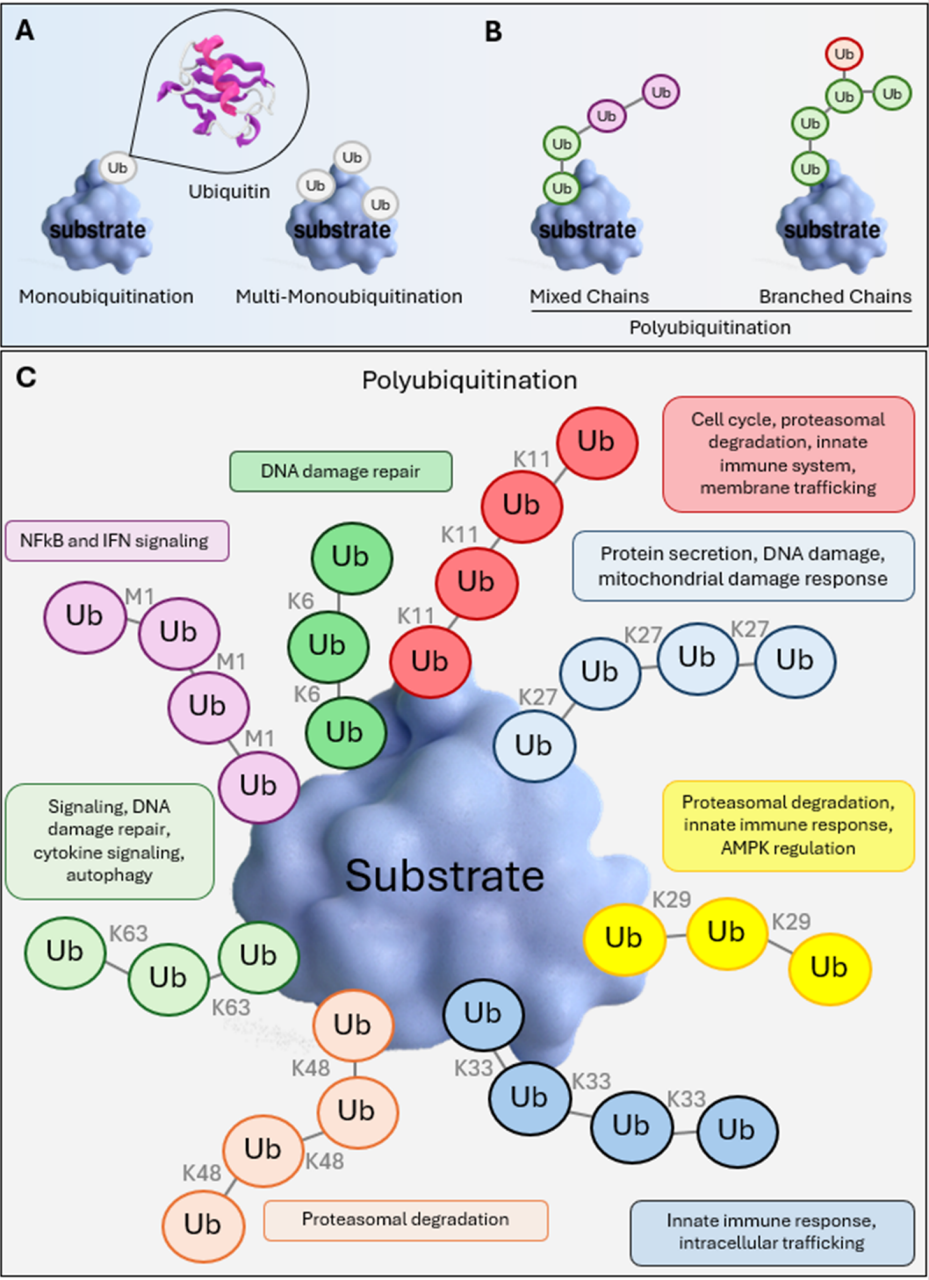
Different types of ubiquitin linkages. Ubiquitin can be attached to the substrate as (A) monoubiquitination and multi-monoubiquitination and (B) polyubiquitination, where (C) different ubiquitin molecules are attached to one another, resulting in the regulation of various cellular processes depending on the residue used for linkage.

### 2.1 E3 Ligases

E3 ligases catalyze the selective attachment of ubiquitin to a lysine, serine, threonine, or cysteine residue of a specific substrate and therefore represent the last step of the enzymatic cascade that marks substrates for proteasomal degradation.^9,105^ The E3 ligases can recognize degradation signals called degrons that are amino acid sequences within a substrate protein that are sufficient for recognition and consequently result in degradation.^106,107^ Importantly, the activity of degrons can be regulated by post-translational modifications such as phosphorylation and hydroxylation or via particular conformational states in the case of cryptic degrons.^106,107^ The E3 ligase von Hippel–Lindau (VHL), for example, only recognizes the degron on its substrate hypoxia-inducible factor when a conserved proline residue is hydroxylated, a process that is abrogated during hypoxic conditions.^108^ Thus, the E3 ligases directly bind to the substrate and, hence, provide regulatory specificity for the UPS.^109,110^ In fact, more than 600 E3 ligases are encoded in the human genome.^5,85,111–115^ A specific E3 ligase may target a subset of proteins with similar structural motifs, and some proteins can be recognized by more than one E3 ligase by distinct degrons.^35^ Overall, E3 ligases can be divided into HECT, Really Interesting New Gene (RING), U-box, and RBR E3 subgroups according to their function and structure (Table 1).

**Table 1:** E3 Ligase Groups with Their Characteristic Domains and Mechanism of Ubiquitin Transfer to Substrates

E3 ligase group	characteristic domain	ubiquitin transfer to substrate
HECT	HECT domain	indirectly from the cysteine residue within the HECT domain
RING	RING domain	directly from E2 to substrate
U-box	U-box domain	directly from E2 to substrate
RBR	RING1, IBR, RING2 domains	indirectly from the cysteine residue within the RING2 domain

The HECT (homologous to the E6AP carboxyl terminus) E3 ligases family is characterized by a common HECT domain, which contains a cysteine residue on which ubiquitin is transferred from an E2 protein.^116,117^ HECT-containing proteins subsequently transfer the ubiquitin attached to their active site cysteine onto the substrate protein. Substrates bind to the N-terminal of the HECT E3 ligase, which differs between members of the HECT E3 ligase family, further classifying this family into three groups: Nedd4 family, HERC family, and others such as HUWE1 and E6-associated protein.^110,117^

RING E3 ligases contain a RING domain and represent the major type of E3 ligases in human cells.^105,118,119^ In contrast to HECT E3 ligases, which transfer ubiquitin to the substrate indirectly, RING E3 ligases bind to E2 proteins via their RING domain and catalyze ligation of ubiquitin to the substrate directly from the E2 conjugating enzyme without an E3-ubiquitin intermediate.^120,121^ Furthermore, RING E3 ligases either act as monomeric RING fingers or multi-subunit E3 ligases, which separate them into two groups. Monomeric RING E3 ligases, such as COP1, murine double minute 2 (MDM2), and TRAF6, have the ability to auto-ubiquitinate in addition to substrate binding and ubiquitination of the substrate.^122^ As the name suggests, multi-subunit E3 ligases are composed of multiple subunits. For example, the Cullin-RING ligases contain the Cullin scaffold (CUL1-9), a RING-box protein at the N terminus (RBX1 or RBX2), an adaptor protein, and a substrate receptor at the C terminus.^123^ The catalytic activity of Cullin-RING ligases is regulated via NEDD8 modification, which enhances E2-ubiquitin recruitment.^124^ APC/C represents another multi-subunit RING E3 ligase and is made of 19 subunits, such as the RING subunit (Apc11) and a Cullin-like subunit (Apc2).^125,126^ The largest E3 ligase complex is formed by the SCF E3 ligase composed of **S**KP1, **C**ullin1, and an **F**-box protein.^127^ The F-box protein functions as a substrate receptor, SKP1 binds to both the F-box protein and Cullin1, and Cullin1 acts as a scaffold bringing the E2 protein close to the substrate by binding to SKP1 and RBX1, which contains the canonical RING domain.^128,129^ SKP1 binds to a set of 69 unique F-box proteins, which recruit different substrates for SCF-mediated ubiquitination.^130^

U-box E3 ligases perform substrate ubiquitination similar to RING E3 ligases, where the E2 protein binds to the approximately 70 amino acid long U-box domain and the ubiquitin is directly transferred to the substrate.^131,132^

RING-IBR-RING (RBR) E3 ligases harbor two RING domains, RING1 and RING2, as well as an in-between-RINGs (IBR) domain.^133^ The RING1 domain recruits the E2 protein, and the RING2 domain contains an active site cysteine, to which ubiquitin is transferred before attaching it to the substrate.^134–136^

### 2.2 Deubiquitinating Enzymes

Ubiquitination of proteins is involved in multiple cellular processes, and, like other post-translational modifications, it is reversible through a process called deubiquitination. The process of deubiquitination is catalyzed by the family of deubiquitinating enzymes (DUBs), which comprises approximately 100 proteins that can be divided into cysteine peptidases and zinc metallopeptidases.^35,137^ In addition to cleaving off ubiquitin from substrate proteins (Figure 1), DUBs can also edit ubiquitin chains and process ubiquitin precursors.^138^ One critical function of DUBs is taking place during proteasomal degradation, where deubiquitination of substrates and the associated recycling of ubiquitin molecules is coupled to substrate binding and transmission to the proteasome.^1,35,139^ Several DUBs have been identified to physically interact with the proteasome, where they remove the polyubiquitin chain from proteins targeted for proteasomal degradation.^140–146^ DUBs also regulate networks of ubiquitin substrates beyond proteasomal degradation, including autophagy, apoptosis, genome integrity, cell cycle, mitochondrial function, signal transduction, and transcription.^147^ DUBs can regulate both stability and activity of substrate proteins and are subject to a complex network of regulation that fine-tunes the activity and specificity of these proteins.^138^ Considering the involvement of DUBs in various cellular pathways and their dysregulation in diseases such as cancer, neurodegenerative diseases, or chronic inflammation, makes them attractive targets for drug discovery.^137^

## 3 Different Types of Targeted Protein Degradation Mechanisms

TPD represents an exciting field where the UPS or lysosomal degradation pathways are employed to target proteins for degradation, serving as a novel tool to study cellular pathways, but also represents a promising therapeutic strategy beyond conventional drug design.^7,148^ Notably, TPD has the potential to expand the “druggable” space and target disease-causing proteins, which have been historically challenging to inhibit with conventional small molecules, thereby creating a new paradigm in drug development.^3,148^ Most TPD strategies rely on the UPS to target intracellular proteins, while lysosome-dependent TPD approaches have the potential to target extracellular proteins, membrane proteins, and protein aggregates.^7^ The different types of TPD focusing on UPS-based approaches are discussed below.

### 3.1 PROTACs

The first PROTAC was introduced in 2001 by Crews and Deshaies groups, which was named Protac-1.^6^ PROTAC is a heterobifunctional molecule that is composed of an E3 ligase-recruiting ligand, a warhead targeting a POI, and a linker connecting the two (Figure 3). Protac-1 was designed to recruit methionine aminopeptidase-2 via ovalicin to the SCF E3 ligase complex for proteasomal degradation via a phosphopeptide derived from IkBα (IPP).^6^ PROTACs form a ternary complex consisting of the PROTAC itself, the POI, and the E3 ligase which results in the E3-mediated ubiquitination of the POI and the subsequent proteasomal degradation via the UPS.^3,7,9,149,150^ Protac-1 was built from both small molecule (methionine aminopeptidase-2 binder) and peptide (phosphopeptide that interacts with the E3 ligase β-TRCP of SCF complex).^6^ An important milestone for PROTACs was the introduction of the first small molecule-based PROTAC in 2008.^151^ Schneekloth et al. designed the small molecule-based PROTAC consisting of a nonsteroidal androgen receptor (AR) ligand (SARM) and a ligand of MDM2 E3 ligase linked via a PEG-based linker to target AR for proteasomal degradation.^151^ Compared to PROTACs containing peptides, small molecule-based PROTACs have the advantage of improved uptake by cells and can be developed into drugs.^152^ For the design of PROTACs, other E3 ligases have been used in addition to MDM2, such as Cereblon (CRBN),^153^ cell Inhibitor of Apoptosis Protein (cIAP),^154^ VHL,^155^ and Cullin E3 ligase adaptor protein SKP1.^156^ A variation from PROTACs are chimeric molecules termed specific and non-genetic Inhibitor of Apoptosis Protein (IAP)-dependent protein erasers (SNIPERs) that recruit IAP ubiquitin ligases to POIs to target them for proteasomal degradation and have been proven successful in numerous target proteins.^157^ In contrast to other PROTACs, SNIPERs degrade not only the POI but also the IAPs themselves, which may be an advantageous feature when targeting cancer cells that regularly overexpress IAPs as a means of cancer therapy resistance by inhibiting apoptosis.^157–159^ Moreover, PHOtochemically Targeting Chimeras (PHOTACs) were developed to harbor a photoswitch in addition to the E3 ligase ligand and POI recruiter (trifunctional molecule) to enable activation of PHOTACs to degrade BET family proteins, including BRD4, via spatiotemporal precision using different wavelengths of light.^160^ These examples further underscore the versatility of PROTAC technology and its potential as a new therapeutic modality.

**Figure 3:**
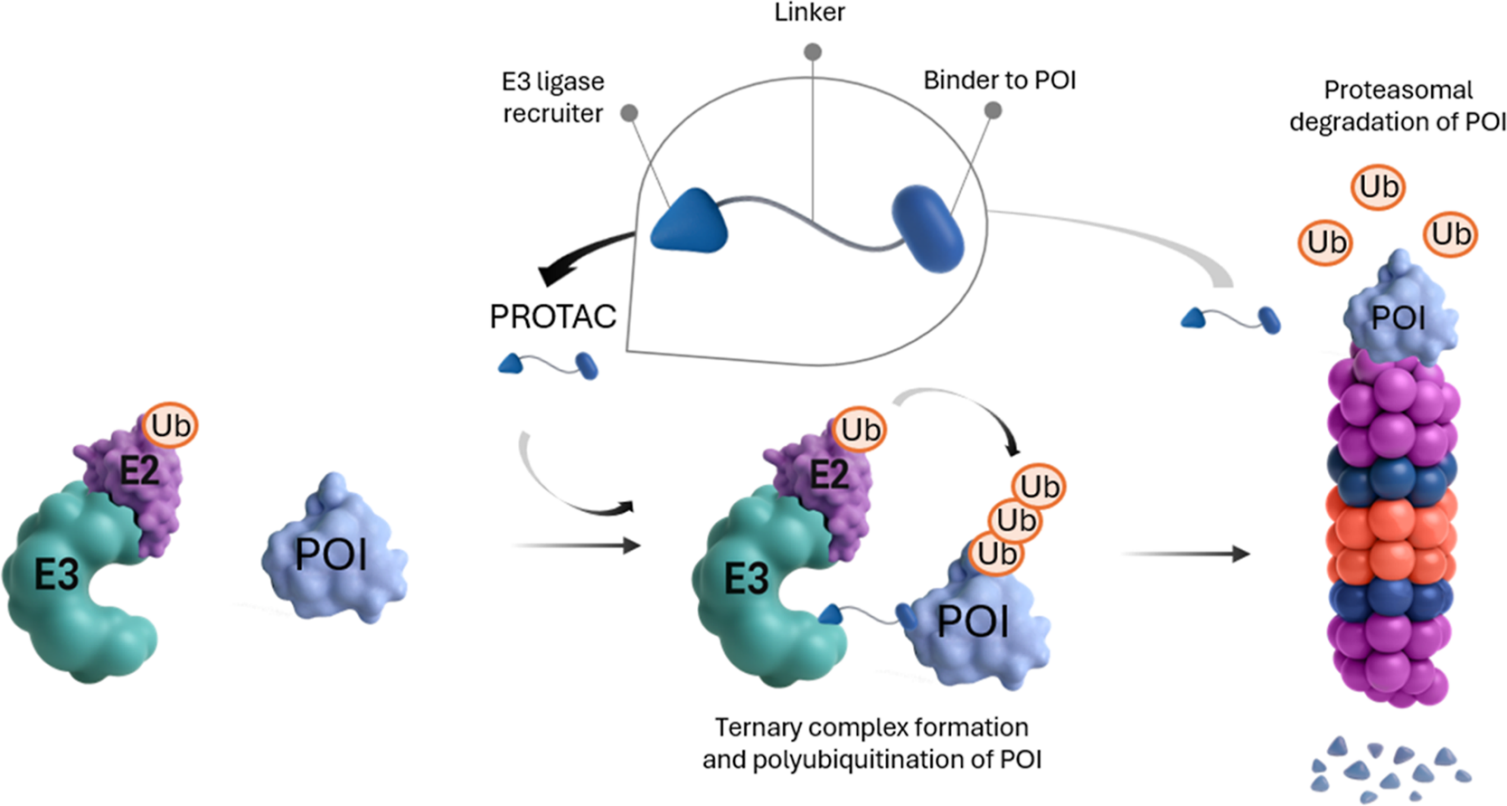
Mechanism of PROTAC-mediated degradation of a POI via the UPS. PROTACs are heterobifunctional molecules that bring a POI and E3 ligase into close proximity to induce polyubiquitination of the POI via the E3 ligase. After iterative ubiquitination steps, the POI is sent for proteasomal degradation. The PROTAC molecule is available for the next round of ternary complex formation, thereby following event-driven pharmacology.

### 3.2 Molecular Glue

Similar to PROTACs, molecular glues also facilitate the formation of a ternary complex with the POI and an E3 ligase that results in ubiquitination and subsequent proteasomal degradation of the POI via the UPS.^161^ However, molecular glues do not bind to both the POI and E3 ligase at the same time as PROTAC but instead achieve ternary complex formation by only binding to either the POI or E3 ligase (more common), which induces or stabilizes the interaction with the other part. Furthermore, molecular glues do not possess a linker, which results in a smaller molecular weight compared to PROTACs, which improves the oral bioavailability and cellular permeability.^7^ Early examples of molecular glues are cyclosporin A (CsA), FK506, and rapamycin that facilitate the complex formation of cyclophilin-CsA-Calcineurin, FKBP12-FK506-Calcineurin, and FKBP12-rapamycin-mTOR (FRB), respectively, and act as immunosuppressants.^162,163^ Thalidomide, lenalidomide, and pomalidomide are examples of molecular glues that act as degraders by inducing binding of the E3 ligase CRBN with its substrate IKZF1/3, which results in the proteasomal degradation of IKZF1/3.^164,165^

Although rational design strategies for molecular glues are emerging, it is more difficult to identify or design molecular glues to degrade a POI compared to PROTACs.^7^ Recently, the Fischer lab conducted diverse screening approaches to identify CRBN-based molecular glue targets in an unbiased manner^166^ as well as defining properties of drug-induced degradation mediated by residues beyond the core structural motif recognized by CRBN.^167^ Furthermore, Petzold et al. conducted computational matchmaking algorithms to perform proteome-wide CRBN target space analyses, which led to the identification of an unexpected plasticity of CRBN, expanded our understanding of target engagement, and created opportunities to expand the target space of CRBN-based molecular glues.^168^

### 3.3 Double-Mechanism Degrader

As the name suggests, double-mechanism degraders harbor two mechanisms to degrade POIs. A small molecule called GBD-9 was reported to target two proteins, Bruton tyrosine kinase (BTK) and G1 to S phase transition 1 (GSPT1) via mechanisms characteristic of both PROTAC and molecular glue.^169^ Yang et al. modulated the length of the BTK PROTAC linker to design GBD-9 that functions as a PROTAC when targeting BTK, but at the same time works as a molecular glue to induce the degradation of GSPT1, which ultimately resulted in a stronger impairment of cancer cell proliferation in multiple cancer cell lines compared to BTK inhibitor ibrutinib.^169^

### 3.4 Tag-Based Technologies

Over the past two decades, several tag-based technologies have been designed to modulate protein homeostasis. Inspired by the plant degradation system based on the plant hormone auxin, Nishimura et al. designed the auxin-induced degron (AID) system in non-plant cells to conditionally modulate protein stability with a small molecule. By tagging a POI with the AID degron and ectopically expressing the F-box family protein TIR1 from *Arabidopsis thaliana* that contains the F-box domain with which it binds to endogenous SKP1 of the SCF E3 ligase complex, POIs can be degraded via the proteasome in the presence of auxin.^170^ Without the need for ectopic expression of additional proteins, small-molecule displacement of cryptic degron can be used to induce conditional protein degradation by fusing a ligand-induced degradation domain, consisting of FK506- and rapamycin-binding protein (FKBP) with a C-terminal 19 amino acid long degron, to the POI.^171^ The presence or absence of a small molecule termed Shield-1 either degrades or stabilizes the target protein, respectively.^171^ Another tag-based system takes advantage of the protein quality control machinery’s ability to detect exposed hydrophobic residues, which are otherwise buried within the core of the protein and represent a hallmark of unfolded proteins, which are consequently degraded by the UPS or autophagy.^172–175^ To mimic the partially unfolded state and thus destabilize a POI, Neklesa et al. designed hydrophobic tags fused to a HaloTag haloalkane reactive linker that forms a covalent bond with a HaloTag fusion protein, which resulted in rapid and robust degradation of diverse target proteins.^176^ In a similar fashion, the same lab developed HaloPROTACs consisting of chloroalkane linked to a hydroxyproline derivative to recruit HaloTag7 fusion proteins and the E3 ligase VHL, respectively, resulting in the proteasomal degradation of the POI.^177^ The dTAG system comprises a technology where POIs are endogenously tagged by CRISPR-mediated knock-in of FKBP12^F36V,^ allowing rapid allele-specific degradation of the POI by recruiting the E3 ligase CRBN to the FKBP12^F36^-tagged POI upon treatment with heterobifunctional degraders linking CRBN ligands to (ortho-) AP1867.^178,179^ To circumvent ligand binding to endogenous FKBP12, which could have confounding biological effects, FKBP12^F36V^ was engineered to harbor a specificity cavity to which the synthetic ligand AP1867 binds with a 1000-fold selectivity over the FKBP12 wild type (WT).^180^ Another innovative system to control protein abundance is called small molecule-assisted shutoff (SMASh); it uses a self-excising degron tagged on target proteins.^181^ The HCV NS3 protease recognition site enables removal of the SMASh tag via internal protease activity, which can be inhibited by protease inhibitors, leading to the degradation of the tagged protein.^181^

### 3.5 Trim-Away

In contrast to the methods described above, Trim-Away is a TPD strategy without the need for prior modification of the genome or mRNA.^182^ Clift et al. designed Trim-Away as a post-translational protein depletion method that is based on antibodies for targeting POIs, which are widely commercially available and bind with high affinity and specificity to their targets. Instead of using the antibody to interfere with protein function by competing with a ligand or binding to an epitope that impairs protein function, Trim-Away utilizes the cytosolic antibody receptor and E3 ligase TRIM21 that binds to Fc domains of antibodies and recruits the UPS to the antibody-bound pathogen, or in the case of Trim-Away, the POI, which results in its proteasomal degradation.^182–185^ TRIM21 further demonstrated to be able to target multimeric proteins such as the nuclear core complex and proteins within biomolecular condensates for proteasomal degradation via the molecular-glue (*S*)-ACE-OH-induced interaction between TRIM21 and NUP98, thereby expanding the applications of TRIM21-based degraders.^186^

### 3.6 Targeted Protein Degradation via Lysosomes

Complementary to PROTACs, which target intracellular proteins for proteasomal degradation, lysosome-targeting chimaeras (LYTACs) were designed to promote degradation of extracellular and membrane proteins, harnessing the endosome–lysosome pathway and thereby gaining access to approximately 40% of encoded proteins that were inaccessible to TPD strategies using the intracellular protein degradation machinery.^187–189^ LYTACs consist of a small molecule or antibody fused to glycopeptide ligands (agonists of the cation-independent mannose-6-phosphate receptor) to facilitate ternary complex formation with the extracellular domain of a membrane or extracellular protein and a lysosome-targeting receptor, which results in the clathrin-mediated endocytosis, protein internalization, and subsequent lysosomal degradation.^187,188^

In addition to LYTAC, bispecific aptamer chimera,^190^ antibody-based PROTAC (AbTAC),^191^ GlueTAC,^192^ autophagy-targeting chimera (AUTAC),^193,194^ autophagosome tethering compound (ATTEC),^195^ AUTOphagy-TArgeting Chimera (AUTOTAC),^196^ and chaperone-mediated autophagy (CMA)-based degrader^197,198^ are innovative strategies that were designed to target proteins for lysosomal degradation.

## 4 Design of PROTACs

The PROTAC technology has matured from proof-of-concept experiments using peptidic ligands in cell lysates^6^ to fully synthetic molecular compounds.^179,199^ PROTACs have successfully been employed against a diverse set of protein classes across multiple subcellular locations and are used in cultured cells, in vivo, and have already entered clinical trials.^5^ With recent advances in the biophysical and structural characterization of PROTACs and the ternary complexes they form with their targets, protein–protein interactions between the E3 ligase and the neo-substrate (the POI) were identified to play a crucial role in PROTAC efficacy.^200–206^

To design a PROTAC, the workflow described by Burslem and Crews can be followed.^5^ PROTACs consist of a ligand to the E3 ligase, a linker, and a ligand to the POI, and all three parts need to be considered when designing a PROTAC. First, a target protein needs to be selected. How PROTACs can extend the druggable proteome will be discussed in more detail in the next sections. Once the target has been chosen, the literature can be searched for available ligands, preferably with known crystal structure or structure–activity relationships to identify a suitable linker attachment location.^5^ It is also feasible to screen small molecule libraries for compounds that can directly bind to the POI using surface plasmon resonance or small molecule microarrays.^207–211^ Different linker lengths, attachment sites, and compositions should be tested, as it was shown that these could impact PROTAC function substantially.^177,212–214^ A few E3 ligases have been employed for the design of PROTACs due to the availability of small molecules to recruit them, including CRBN and VHL, which have been the workhorses of PROTACs.^5,179,215^ CRBN-based PROTACs comprise the most-studied group of PROTACs, with many already entering clinical trials.^216^ Moreover, ligands to cIAP have been used which degrade the E3 ligase itself via auto-ubiquitination.^217^ Nutlin has been employed to recruit MDM2,^151,218^ and specific recruiters to DCAF16 have been described for the nuclear-restricted degradation of target proteins.^219^ It is important to note that having a good prior knowledge of ideal chemotype–target pairs can accelerate the hit-to-lead process dramatically when starting a protein degrader discovery project.^220–226^

The importance of E3-PROTAC-POI ternary complex formation and protein–protein interactions between E3 ligase and the neo-substrate was highlighted recently. Bondeson et al. showed how PROTAC designed with a promiscuous kinase ligand binding over 50 kinases resulted in degradation of only a subset of the targets sparing kinases with unstable ternary complexes.^200^ Interestingly, they did not observe a correlation between the affinity of the POI to the PROTAC warhead and level of degradation mediated by PROTAC. Instead, the best predictor of target protein degradation potency was the POI’s ability of stable ternary complex formation with the E3 ligase and PROTAC.^200^ This is in line with the structural basis of the ternary complex that was resolved for the degrader MZ1 with its target BRD4 and E3 ligase VHL.^201^ Gadd et al. demonstrated specific intermolecular interactions that dictated the specificity of the degrader to its target, thus exemplifying how de novo interactions between POI and E3 ligase that are induced by PROTAC are regulating preferred recruitment of the target protein into a stable ternary complex, which allows selective target protein degradation. Moreover, cooperative protein–protein interactions between POI and E3 ligase could also benefit potency^200^ and selectivity.^227^ In this regard, the linker composition, linker attachment point, and length can have a profound effect on PROTAC potency, likely due to steric interactions that regulate the PROTAC’s ability to simultaneously bind to the POI and E3 ligase, which can result in positive or negative binding cooperativity.^177,228^ It is generally beneficial to initially design PROTACs with slightly longer linkers that can then be gradually shortened until negative effects on activity are observed.^179^ However, linkers that are extended too far can also negatively impact target protein degradation.^213^ Overall, the linker can be modulated to affect the molecular weight of PROTAC by adjusting linker length,^229^ alter PROTAC properties,^230^ contribute to solution conformation,^231^ and influence the degradation efficacy^232^ and presentation of the POI in the zone of ubiquitination.^3^

The examples mentioned above are limited to the specific scaffold and protein target discussed and may not hold true for other targets. Donovan et al. investigated the degradability of the kinome, where they concluded that cellular target engagement and stable ternary complex formation did not predict degradation success, but protein kinases showed differences in tolerance for linker design changes that were, however, inconsistent.^220^ Overall, the authors recommended data-driven approaches and highlighted the complexity of mechanisms of TPD that need to be systematically examined to gain a better understanding of degradation activity prediction and the rational design of TPD molecules.

After iterative rounds of screening and synthesis of a sufficiently potent PROTAC that degrades its targets, thorough PROTAC validation and characterization should be performed.^5^ The target protein mRNA levels can be tested via qPCR to ensure that the PROTAC is acting on the protein at the post-translational level and not by decreasing the mRNA.^199^ The pan-JAK ATP-competitive inhibitor PF-956980, for example, was shown to reduce protein levels of its targets JAK2 and JAK3 through downregulation of their mRNA.^233^ Next, experiments to confirm involvement of the UPS should be performed, which can be achieved by inhibiting the proteasome using inhibitors such as bortezomib,^234^ MG132,^235,236^ and NEDD8-activating enzyme inhibitor MLN-4924 to confirm involvement of an active E3 ligase, as Cullin ligases get activated via neddylation.^237^ The cotreatment with either of these compounds should stabilize target protein levels. In addition, quantitative proteomics would allow the identification of potential off-target effects and selectivity data of PROTACs.^5^ Importantly, the decrease of other proteins could also be a biological consequence of the POI loss, as is the case with c-MYC when BRD4 is degraded.^179,235^

Furthermore, to ensure that the ligand binding alone without the recruitment of the E3 ligase does not destabilize the POI, as is observed via hydrophobic tagging or selective estrogen receptor degrader fulvestrant, inactive PROTACs should be included as negative controls.^176,238–240^ Inactivating a PROTAC compound can be achieved by discrete modification of the ligand recruiting the E3 ligase, for example, for the VHL ligand through a simple inversion of stereocenters.^213^

### 4.1 Advantages and Disadvantages of PROTACs

One advantage of PROTACs arises from their catalytic mechanism, which follows event-driven pharmacology where one PROTAC molecule results in multiple ubiquitination events.^2,199^ Even 10% occupancy of the E3 ligase was shown to be sufficient to effectively degrade a POI,^219^ and low-affinity ligands can be employed.^203,241^ Moreover, many benefits from using PROTACs stem from their small-molecule nature, which makes their utilization comparable to classic inhibitors without the need for transfection reagents, viruses, or specific culture conditions.^5^ In line with this is the opportunity to use PROTACs to modulate POI protein levels in a dose-dependent manner, which is difficult to achieve with genetic perturbation strategies.^5^ Due to their unique mechanism of action, PROTACs had superior activity compared to classic inhibitors in some instances. For example, compared to the traditional inhibitors, PROTACs targeting histone deacetylases (HDACs) demonstrated improved selectivity, more effective impairment of cancer cell growth, overcoming drug resistance, and impairment of target protein functions independent of enzymatic activity.^228,242–249^ Furthermore, once PROTACs have been established in one system, they can be easily applied to others, allowing screening of protein function across different cell lines or other challenging contexts such as primary cells derived from patients.^212^ In keeping with this, PROTACs were effective in vivo without prior genetic perturbation of the animal, and early data also support clinical efficacy; however, physicochemical properties may have to be optimized.^178,179,199,250,251^ Interestingly, due to their heterobifunctional nature, PROTACs do not adhere to Lipinski’s rule of 5, which describes a physicochemical property guideline for oral bioavailability of small molecules in humans^3,252–255^ and exhibit more favorable pharmacokinetic properties than anticipated given their molecular weights. Another important advantage of PROTACs is the rapid kinetics, where protein degradation can already be achieved in 1 h, which allows studying the acute consequences of a target protein loss rather than observing effects that could be a consequence of cellular reprogramming during selection for cells that survive the loss of the protein via genetic perturbation.^5^ At the same time, PROTACs can be withdrawn from the cells, which allows the restoration of target protein levels, although even after PROTAC washout, some PROTAC may continue to reside in cells.^213^

Despite all of the benefits that were discussed above, there are several disadvantages that provide obstacles to the widespread use of PROTACs. The discovery phase can be rather lengthy since PROTACs require a ligand to the POI to be converted into a potent PROTAC, which is not always straightforward.^5^ Additionally, as with other techniques, PROTACs are prone to off-target effects, which is why it is critical to use quantitative proteomics to assess selectivity profiles of the PROTAC.^256^ CRBN-based PROTACs in particular should be used with care since the IMiD component used to recruit CRBN can lead to the degradation of zinc-finger neo-substrates.^257–260^ Another phenomenon that is associated with PROTACs is the hook effect, where increasing concentrations of PROTACs do not always lead to a greater degree of degradation.^261^ This can be explained by the formation of unproductive dimers that hinder the active ternary complex formation that is necessary for target protein degradation, which has to be accounted for when thinking about dosing regimens and pharmacokinetics and -dynamics.^5^ Lastly, the current form of PROTAC technology is not able to access every protein in each subcellular compartment, such as multi-pass transmembrane proteins, but this may be achieved in the future by leveraging E3 ligases that are localized to these subcellular compartments.^5,262^

In addition to drug efficacy, drug accessibility, linked to drug manufacturing technologies and costs, is a critical aspect when it comes to the overall impact a drug can have on patients’ lives. Biologics, medicines derived through biological processes from living cells are complex, heterogeneous structures that are sensitive to a given manufacturing process and the starting materials and include monoclonal antibodies, hormones, vaccines, and gene and cellular therapies.^263^ Over the past years, biologics have been responsible for more exciting strides in healthcare development compared to small molecules. Prominent examples of biologics that have revolutionized cancer therapy are CAR-T cell therapy, which is the leading cancer cell therapy in oncology clinical trials,^264^ as well as PD-L1 and PD-1 blocking antibodies that induce antitumor T cell responses.^265^ Moving beyond oncology, the GLP-1-based therapies semaglutide or tirzepatide, used for the treatment of obesity and type 2 diabetes, are GLP-1 receptor agonists,^266^ which have both been ranked among the top 10 drugs by sales in 2024 (Ozempic/semaglutide 17.5 billion $US ranked #2; Mounjaro/tirzepatide 11.5 billion $US ranked #8).^267^ The top selling drug in 2024 was Keytruda, a monoclonal antibody blocking PD-1, accounting for 29.5 billion US$ in sales in 2024.^267^ While these biologics have undoubtedly made a huge impact in patient outcomes, they do come with significant costs that make them inaccessible to many patients with lower incomes. For a daily dose of biologic, the price is on average 22 times higher than that of a small molecule.^263^ Chemically synthesized small molecules, in contrast to biologics, are more economically sustainable and more accessible to patients.^263^ Traditional small molecules still account for 90% of global sales in today’s drug market;^263^ however, their occupancy-driven pharmacology is restricted to binding to well-defined pockets within proteins, which makes approximately 80% of the proteome inaccessible due to a lack of a chemical ligand.^268^ Only 3% of the proteome has been targeted by approved, relevant therapies,^268,269^ which provides a unique opportunity for induced proximity-based therapeutics such as PROTACs to overcome existing limitations and access the “undruggable” proteome.^270^ Importantly, since small molecule PROTACs can be chemically synthesized, PROTACs are expected to be more accessible to patients with lower drug prices compared to biologics.

#### 4.1.1 Strategies to Improve Physicochemical Properties of PROTACs

In addition to the above-mentioned limitations, PROTACs are prone to poor physicochemical properties due to their large molecular weight and high hydrophobicity, which limits the bioavailability, impairs the therapeutic efficacy, and results in suboptimal pharmacokinetics. Taking this into consideration, several strategies have been employed recently to improve physicochemical characteristics and the in vivo efficacy of PROTACs. Novel approaches aim to enhance non-specific biodistribution, suboptimal solubility, the hook effect, limited permeability, and low bioavailability.^271–276^

One such approach utilizes click-release PROTAC prodrugs that can be activated within the target cells.^277^ Lebraud et al. designed a bio-orthogonal click combination PROTAC prodrug, in which the heterobifunctional molecule is assembled intracellularly from two small precursors utilizing a CRBN-recruiting derivative of thalidomide tagged with tetrazine that reacts with the POI ligand harboring a *trans*-cyclo-octene.^278^ The advantage of the click-formed proteolysis targeting chimeras (CLIPTACs) is that these two smaller precursors facilitate improvements in water solubility, tissue permeability, and target selectivity. Similarly, Huang et al. designed a VHL-based PROTAC prodrug containing 4-(vinyloxy) benzyl carbonate that cages the PROTAC until its activation via an inverse electron-demand Diels–Alder reaction in the presence of a 3,6-dimethyl-1,2,4,5-tetrazine.^279^ A cancer biomarker-activating PROTAC prodrug was developed by Chang et al. that uses the presence of integrin αvβ3 biomarker found in cancer cells to selectively activate the PROTAC in the cancer cell but not in non-transformed cells. The inactive PROTAC prodrug harbors a VHL ligand conjugated to a biorthogonal *trans*-cyclooctene group, which can be activated by the integrin αvβ3-targeting tetrazine-modified RGD peptide.^280^

Instead of utilizing click-release chemistry to activate PROTAC prodrugs within target cells, a group of PROTAC prodrugs was designed to target folate receptor α, often overexpressed in tumors and an attractive anti-cancer drug target, biomarker, and marker for selective anti-cancer drug delivery.^281^ Folate-conjugated PROTAC prodrugs were designed to selectively target tumor cells, which overexpress folate receptor α, thereby decreasing the potential toxicity of PROTACs in normal cells due to the off-tissue on-target degradation of the target protein.^282,283^ Other PROTAC prodrug strategies to selectively target tumor cells include radiation-responsive PROTAC prodrugs, in which the PROTAC is only active in response to radiotherapy,^284,285^ tumor microenvironment-responsive PROTAC prodrugs, where PROTAC activation relies on the overexpression of an enzyme or protein in the tumor (microenvironment) compared to normal tissue,^285–288^ hypoxia-responsive PROTAC prodrugs,^289–293^ and ROS-responsive PROTAC-prodrugs.^294,295^

Several PROTAC prodrugs were designed to be under the control of light and are termed PHOTACs. Photo-caged PROTAC prodrugs^296–299^ are compounds that are activated with the appropriate wavelength of light via incorporation of photocleavable caging groups that impair binding to the E3 ligase or POI, whereas photo-switchable PROTAC prodrugs^160,300–302^ use ligands that, depending on the presence or absence of a certain wavelength, can switch between active and inactive states.^303,304^

Another strategy to improve physicochemical properties of PROTACs, such as low aqueous solubility and poor pharmacokinetics, which limit in vivo applications, uses the conjugation of the PROTAC to an antibody, a degrader-antibody conjugate, where the antibody facilitates delivery of the PROTAC to the desired target cell.^305–311^

Lastly, nano-PROTAC polymers, which utilize nanosized drug delivery systems, have the potential to improve the blood circulation time of PROTACs, enhance tumor distribution, and increase cellular uptake of PROTACs.^312–326^

Further emphasizing that PROTACs can overcome their limitations in physicochemical properties are examples of degraders, such as the LRRK2-targeting PROTACs ARV-102 and XL01126, that have demonstrated to pass the blood–brain barrier to treat diseases in the central nervous system.^327–331^

Taken together, several innovative strategies have been discovered to improve physicochemical properties such as decreased membrane permeability, poor water solubility, and high polarity, as well as on-target off-tissue effects of PROTACs. In addition to optimizing the chemical structure of PROTACs, PROTAC prodrugs, PROTAC-antibody conjugates, and nano-PROTACs have demonstrated promising results in overcoming traditional limitations and may improve clinical applications.

### 4.2 Optimizing the E3 Selection Process

The human genome encodes for only two ubiquitin E1 enzymes (UBA1 and UBA6), roughly 40 E2 proteins, but more than 600 E3 ligases, highlighting how specificity within the UPS is mainly regulated via E3 ligases and the potential for innovative and disease-specific PROTAC design.^5,85,111–115^ However, the type of ubiquitination catalyzed by the E3 ligases remains poorly understood for numerous E3 ligases, and, in addition, less than half of the E3 ligases are believed to be functioning within the UPS, which is an important aspect to be considered when selecting an E3 ligase for PROTAC design.^112,332^ There is enormous therapeutic potential with thousands of PROTACs that have been designed to target a diverse set of proteins.^333,334^ However, of the more than 600 that are encoded in the human genome, only a few E3 ligases have been used for the design of PROTACs (Table 2).^111–113,115^ The E3 ligase is a crucial component of PROTACs and has the potential to reduce on-target toxicities.^3,335–337^

**Table 2:** Examples of E3 Ligases Used in PROTACs^a^

E3 moiety	native substrate	ligand	references
VHL	hydroxylated HIF-1α	VHL peptidomimetics	^199^,^215^ and^348^
CRBN	glutamine synthetase MEIS2	IMiDs	^179^ and^235^
MDM2	p53	(idasa-)nutlin	^151^ and^218^
β-TRCP	β-catenin	phosphorylated peptide	^6^
cIAP	second mitochondrion-derived activator of caspases (SMAC)	bestatin esters, MV-1, LCL161 (SMAC mimetics)	^349^
RNF4	poly-sumoylated proteins	CCW-16 (covalent fragment)	^350^
RNF114	p21	nimbolide (natural product)	^351^
DCAF16	unknown	KB02 (covalent fragment)	^219^
KEAP1	nuclear factor erythroid 2-related factor-2 (Nrf2)	bardoxolone methyl	^352^ and^353^
SKP1	p27, cyclin E	EN884	^156^

a Modified from the original citation.^3,5,347^

The expression levels of the E3 ligase in the target context are important, and selecting a higher expressed E3 ligase may improve function and minimize protein degradation in non-target tissues with low E3 ligase expression.^3,335^ E3 ligases with unique tissue-selective expression profiles present desirable therapeutic opportunities as they are expected to reduce unwanted toxicities.^332^ CDC20, for example, is a substrate receptor of the APC and is highly expressed and essential in numerous cancer types, whereas it shows very low to no expression in around 70% of non-malignant tissues.^332,338^ Selective E3 ligase expression patterns were beneficial for BCL-XL targeting PROTAC development. BCL-XL-dependent leukemia cells express relatively high levels of VHL compared to those of platelets. Consequently, DT2216, which recruits VHL to target BCL-XL, achieved potent anti-tumor activity with considerably less on-target and dose-limiting thrombocytopenia.^339,340^ Moreover, specific subcellular localization of the POI and E3 ligases can be leveraged when designing PROTACs and choosing an E3 ligase. DCAF16, for example, is a nuclear E3 ligase that selectively degrades its target in the nucleus when incorporated in a PROTAC.^219^

In addition, optimizing E3 ligase selection could counteract acquired drug resistance via mutations at the E3 ligase loci.^3,336^ Relying on non-essential E3 ligases such as CRBN and VHL poses a particular risk when used in cancer, as cancer cells could quickly mutate and evade PROTAC mechanisms without impairing their viability through loss of the non-essential E3 ligase function.^3,341^ Emerging resistance was observed in multiple cases that relied on CRBN- and VHL-based degraders, where mutations and downregulation of the UPS machinery were identified.^342–346^ VHL-based resistance mechanisms can result from the loss of CUL2 and depletion of CRBN itself, or its E2 enzyme UBE2G1 can lead to resistance to CRBN-based PROTACs.^344,345^ Thus, selecting E3 ligases that are dependent on a particular cancer could circumvent resistance mechanisms to a greater extent.

In order to expand the E3 ligase toolkit for PROTACs, Liu et al. have focused on seven aspects guiding E3 ligase selection for a given target and utilized large-scale data resources to analyze an assembled comprehensive E3 ligase list: (1) chemical ligandability of E3 ligase, (2) expression patterns, (3) protein–protein interactions, (4) structure availability, (5) functional essentiality, (6) cellular localization, and (7) protein–protein interaction interface.^354^ The generated database will serve as a useful tool for future PROTAC design and E3 ligase selection and may provide a step toward fully leveraging the diversity of E3 ligases for improved and disease-tailored PROTACs.

## 5 PROTACs as New Therapeutic Modality

### 5.1 PROTACs in Clinical Trials

Since the first report on PROTAC in 2001,^6^ the technology has gained tremendous interest from both academia and industry. Some of the excitement in the TPD field stems from observations that PROTACs function more effectively in abrogating target protein functions compared to inhibitors. This is the case for BRD4-targeting PROTACs^179,235,355^ which is one of the most commonly used target proteins for PROTACs^356^ and serves as an indicator for technical advances.^160,219,350,351^ Beyond the potential of TPD for drug development, PROTACs have enabled numerous discoveries in biology and serve as excellent tools to study protein function.^5^ According to PROTAC-DB, an online repository of PROTACs, more than 6000 PROTACs have been designed to date to degrade a variety of targets,^333,334^ emphasizing that PROTAC is a rapidly growing field that caught the interest of numerous investigators across different scientific disciplines.

Several biotech and pharmaceutical companies are actively working on PROTACs and have disclosed preclinical and early clinical development programs.^3^ The first studies were conducted on degrading the hormone receptors AR^357^ and ER^358^ using orally bioavailable PROTACs,^359^ which emphasizes the advantage of PROTACs over therapeutic approaches based on RNAi^360^ but also traditional selective estrogen downregulators (SERDs).^361^ Both PROTACs designed against the AR and ER outperformed other available compounds,^358,362–364^ which supported the decision to enter clinical trials. This milestone in 2019 was accompanied by excitement in the TPD field when these first PROTACs designed by Arvinas entered clinical trials to target AR (ARV-110, NCT03888612)^365^ and ER (ARV-471, NCT04072952).^366^ Both degraders moved to phase II trials in 2020 and 2021 after providing the first clinical proof-of-concept in 2020 that PROTACs can be used against these well-characterized cancer targets in prostate and breast cancer. Furthermore, four crucial questions for the TPD field were addressed, namely, (1) do heterobifunctional degraders have drug-like properties? (2) Are PROTACs safe to use in humans? (3) Is the mechanism of action against the target protein as expected? and (4) Will they result in therapeutic effects?^3^ Early clinical profiles of ARV-110 and ARV-471 in patients demonstrated well-tolerated safety with effective doses that pointed toward clinical efficacy.^3,365,366^ More importantly, ARV-471 further progressed into phase III clinical trials in 2023, and after providing positive results from the VERITAC-2 trial^367^ (VERITAC-2 ClinicalTrials.gov number, NCT05654623), Arvinas and Pfizer have just recently submitted a New Drug Application (NDA) with the FDA for ARV-471 for the treatment of patients with ESR1-mutated, ER+/HER-advanced or metastatic breast cancers.^368^ This is truly an exciting milestone for the TPD field, marks the start of a new PROTAC era, and highlights the growing impact of this technology for drug discovery in the future.

Currently, several PROTACs, as well as other TPD molecules like molecular glues, have entered clinical trials, with many more expected to follow (Table 3). This first wave of degraders in the clinic mostly focused on well-established disease-targets with solid characterization of biological and biochemical properties, where data show clinical efficacy of the target inhibition.^3^ This is an important step to show that PROTACs are capable of degrading these well-known targets and establishing PROTACs as a new therapeutic modality. The early success of TPD compounds in the clinic is encouraging the field to now go after proteins that have been historically difficult to target with conventional drug design.^3^

**Table 3:** PROTACs in Clinical Trials Sorted by Drug Target^a^

drug	company	target	disease context	route	clinical phase	NCT identifier	key reference
vepdegestrant (ARV-471)	Arvinas/Pfizer	ER	ESR1-mutated ER+/HER2-advanced or metastatic breast cancer	oral	NDA		^367^
SIM-0270	Simcere Pharma	ER	ER+/HER2-advanced breast cancer	oral	III	NCT06680921	^369^
HRS-1358	Jiangsu Hengrui	ER	breast cancer	oral	I/II	NCT06555068	
						NCT06679036	
AC682	Accutar Biotech	ER	ER+/HER2-breast cancer	oral	I (term.)	NCT05489679	^370^
						NCT05080842	
AC699	Accutar Biotech	ER	ER+/HER2-locally advanced or metastatic breast cancer	oral	I	NCT05654532	^371^
HP-568	Hinova Pharmaceuticals	ER	ER+/HER2 advanced breast cancer	oral	I/II	NCT06757335	^372^
CC-94676/BMS-986365	Celgene/Bristol Myers Squibb	AR	metastatic castration-resistant prostate cancer (mCRPC)	oral	III	NCT06764485	^373^
ARV-110	Arvinas	AR	mCRPC	oral	II	NCT03888612	^357^ and^365^
ARV-766	Arvinas/Novatis	AR	metastatic PC	oral	I/II	NCT05067140	^374^
HP518	Hinova	AR	mCRPC	oral	I/II	NCT06155084	^375^
HRS-5041	Jiangsu Hengrui	AR	advanced PC	oral	I/II	NCT06568094	
						NCT06738745	
AC-176	Accutar Biotech	AR	mCRPC	oral	I (term.)	NCT05241613	
						NCT05673109	
QLH12016	Qilu Pharmaceutical	AR	mCRPC	oral	I	NCT05973149	
GT-20029	Kintor Pharma	AR	androgenetic alopecia	topical	II	NCT06692465	
AH-001	AnHorn Medicines	AR	androgenetic alopecia	topical	I	NCT06927960	^376^
SHR-3591	Jiangsu Hengrui	AR	mCRPC	oral	I		^377^
BGB-16673	BeiGene	BTK	relapsed/refractory chronic lymphocytic leukemia or small lymphocytic lymphoma	oral	III	NCT06973187	^378^
						NCT06970743	
						NCT06846671	
ABBV-101	AbbVie	BTK	B-cell malignancies	oral	I	NCT06887010	^379^
						NCT05753501	
AC-676	Accutar Biotech	BTK	R/R B-cell malignancies	oral	I	NCT05780034	^380^
HSK-29116	Haisco	BTK	R/R B-cell malignancies	oral	I	NCT04861779	^381^
NX-2127	Nurix Therapeutics	BTK and IKZF1/3	R/R B-cell malignancies	oral	I	NCT04830137	^382^
NX-5948	Nurix Therapeutics	BTK	R/R B-cell malignancies	oral	I	NCT06717269	^383^
						NCT05131022	
						NCT06593457	
						NCT06691828	
TQB3019	Chia Tai Tianqing Pharmaceutical	BTK	advanced malignant tumors	oral	I	NCT06943677	^384^
UBX-303061	Ubix Therapeutics	BTK	relapsed/refractory B-Cell malignancies	oral	I	NCT06590961	
BMS-986458	Bristol Myers Squibb	BCL-6	relapsed/refractory non-Hodgkin lymphomas	oral	I/II	NCT06090539	^385^
ARV-393	Arvinas	BCL-6	R/R NHL	oral	I	NCT06393738	^386^
DT-2216	Children’s Oncology Group/Dialectic Therapeutics	BCL-XL	relapsed or refractory solid tumors and fibrolamellar carcinoma	intravenous	I/II	NCT06620302*	^339^
PRT3789	Prelude Therapeutics	SMARCA2	advanced or metastatic solid tumors with a SMARCA4 mutation	intravenous	II	NCT06682806	^387^
PRT7732	Prelude Therapeutics	SMARCA2	advanced or metastatic solid tumors with a SMARCA4 mutation	oral	I	NCT06560645	^388^
RNK-05047	Ranok Therapeutics	BRD4	advanced solid tumors/DLBCL	intravenous	I/II	NCT05487170	
MT-4561	Mitsubishi Tanabe Pharma America	BRD4	advanced solid tumors	intravenous	I/II	NCT06943521	
CFT8634	C4 Therapeutics	BRD9	advanced or metastatic synovial sarcoma and SMARCB1-null tumors	oral	I (term.)	NCT05355753*	^389^
FHD-609	Foghorn Therapeutics	BRD9	advanced synovial sarcoma or advanced SMARCB1-loss tumors	intravenous	I (term.)	NCT04965753*	^390^
CFT1946	C4 Therapeutics	BRAF^V600E^	BRAF^V600^ mutant solid tumors	oral	I	NCT05668585	^391^
CG001419	Cullgen	NTRK	solid tumors	oral	I	NCT06636500	
ASP-3082	Astellas Pharma	KRAS^G12D^	advanced solid tumors	intravenous	I	NCT05382559	^392^
ASP4396	Astellas Pharma	KRAS^G12D^	solid tumors	intravenous	I	NCT06364696	^393^
HSK-40118	Haisco	EGFR	NSCLC with EGFR mutation and solid tumors with BRAF^V600^ mutation	oral	I	NCT06050980	
						NCT06536400	
BG-60366	BeiGene	EGFR mutant	EGFR mutant non-small cell lung cancer	oral	I	NCT06685718	
CFT8919	Betta Pharmaceuticals	EGFR L858R	advanced NSCLC	oral	I	NCT06641609	
KT-253	Kymera Therapeutics	MDM2	high grade myeloid malignancies, acute lymphocytic leukemia, lymphoma, solid tumors	intravenous	I	NCT05775406	^394^
KT-333	Kymera Therapeutics	STAT3	refractory lymphoma, large granular lymphocytic leukemia, solid tumors	intravenous	I	NCT05225584	^395^
KT-621	Kymera Therapeutics	STAT6	atopic dermatitis	oral	I	NCT06945458	
						NCT06673667	
KT-413	Kymera Therapeutics	IRAK4	R/R B-cell NHL	intravenous	I	NCT05233033	^396^
KT-474	Kymera Therapeutics	IRAK4	moderate to severe hidradenitis suppurativa	oral	II	NCT06028230	^397^
						NCT06058156	
BGB-45035	BeiGene	IRAK4	moderate to severe active rheumatoid arthritis		II	NCT07100938	
LT-002-158	Leadingtac Pharmaceutical	IRAK4	hidradenitis suppurativa and atopic dermatitis	oral	I	NCT06082323	^398^
AUTX703	Auron Therapeutics	KAT2A, KAT2B	relapsed/refractory AML and MDS	oral	I	NCT06846606	^399^
NKT-3964	NiKang Therapeutics	CDK2	advanced/metastatic solid tumors	oral	I	NCT06586957	
BTX-9341	Biotheryx	CDK4, CDK6	advanced and/or metastatic breast cancer	oral	I	NCT06515470	^400^

a Modified from the original citation^368^ and data collected from clinicaltrials.gov (last updated October 22, 2025). All ongoing clinical trials are conducted with adult participants. Trials marked with an asterisk following the NCT identifier number accept pediatric participants (under the age of 18).

### 5.2 Resistance Mechanisms and PROTAC-Based Therapies

PROTACs that are successfully employed in clinical trials demonstrate how TPD-based therapies are a promising strategy to overcome acquired resistance mechanisms to existing small molecules inhibitors. One example is the AR as a target in prostate cancer where current treatment strategies are surgical removal of the prostate and androgen deprivation therapy. Antiandrogen therapies may initially work well; however, resistance mechanisms occur frequently that can switch the AR antagonists into partial agonists. Consequently, the sensitivity of the AR increases toward other steroid hormones, which ultimately contributes to the development of lethal and to date incurable castration-resistant prostate cancer.^401^ AR degraders provide a promising strategy to overcome these resistance mechanisms, as they result in the elimination of the protein and are unaffected by mutations that convert an antagonist into an agonist. This is also reflected in the numerous clinical trials and AR degraders in development including BMS-986365, ARV-110, and ARV-766 (Table 3). ARV-766, for example, demonstrated a broad mutational coverage and was able to robustly degrade WT and clinically relevant AR mutants such as L702H, H875Y, and T878A.^374^

As briefly mentioned in previous sections, cancer cells have also developed resistance mechanisms toward PROTACs, which will be an important consideration in the field with many PROTACs progressing in clinical trials.^402^ Upregulation of the drug efflux protein MDR1 has been shown to result in resistance mechanisms to multiple PROTACs targeting KRAS or pathway-related proteins such as MEK, BRD4, CDK9, and FAK. This resistance mechanism could be overcome with simultaneous treatment with MDR1 inhibitor or genetic perturbation of *ABCB1*.^403^ Additionally, E3 ligases that are non-essential in a certain tumor type are prone to resistance mechanisms via loss-of-function mutations in the E3 ligase gene, as reported for CRBN and VHL.^345,346,402^ Shirasaki and colleagues performed genome-scale CRISPR-Cas9-based gene editing studies to identify genetic regulators of tumor cell sensitivity to different classes of oncoprotein degraders.^346^ Resistance in myeloma cells toward CRBN- and VHL-based degraders of different targets was primarily governed by prevention rather than adaptation to oncoprotein degradation. Deletion of CRBN itself or its Cullin-RING ligase complex decreased responsiveness of tested cancer cells toward CRBN-based degraders. A similar observation was made for VHL-based degraders with top hits of the VHL itself and complex members. Interestingly, no cross-resistance mechanisms were observed following sequential treatment of degraders employing different E3 ligases, which may be an avenue to prevent or delay resistance in addition to using E3 ligases that are dependencies in the cancer cell.^346^ In multiple myeloma patients treated with thalidomide and its derivatives, clinical resistance was observed via decreased expression or alternative splicing of CRBN.^404–406^ In addition to downregulation of CRBN, mutations in the CRBN protein involving the C-terminal fragment containing the IMiD binding domain presumably affect drug binding.^260,407,408^ As the field is only beginning to explore the clinical potential of PROTAC-based therapies, it is likely that more resistance mechanisms will emerge. Nevertheless, current data suggest utilizing E3 ligases that are required for cancer cell survival as well as sequential treatment with PROTACs using different E3 ligases or combination treatments, which may be strategies to delay acquired resistance mechanisms.

### 5.3 Opportunities for PROTACs in Pediatric Cancer

TPD is a promising treatment avenue for pediatric cancers. Sarcomas, for example, are rare in adults only comprising 1% of all adult malignancies but account for up to 15–20% of pediatric cancers.^409,410^ Sarcomas are often aggressive diseases with high rates of recurrence and incidence of metastasis, which do not respond well to conventional therapeutic interventions.^411,412^ Consequently, cure rates and patient prognoses remain unacceptably low. Incorporation of targeted therapy into treatment regimens have been limited by acquired resistance mechanisms, limitation of executing subtype-/biomarker-specific trials due to rarity of disease, absence of actionable mutations in sarcomas, and restrictions in the chemical composition of existing inhibitors.^413^ Many childhood cancers, including sarcomas, are characterized by chromosomal translocation that result in chimeric fusion proteins,^414,415^ which often occur in genomic backgrounds with few co-occurring genetic alterations^416–421^ and are therefore thought of as the driving oncogenic event in these tumors. The fusion proteins frequently involve genes important in transcriptional and chromatin regulatory mechanisms, which result in aberrant developmental programs that lead to transformation.^414^ Although these tumor-specific fusion proteins represent attractive therapeutic targets, except for a few fusion proteins harboring kinase domains (Gleevec targeting BCR-ABL),^422^ they have been challenging to target with conventional small molecule design due to the lack of distinct enzymatic pockets, as many fusion proteins are TFs.^414^ Thus, pediatric cancers, such as sarcomas, represent diseases that could benefit from TPD-based therapies.

Synovial sarcoma, for example, primarily affects young men under the age of 30 with around 1000 cases annually. A key driver of disease is the tumor-specific fusion protein SS18-SSX,^423–427^ which relies on the BAF complex to drive cell growth.^428–431^ The BAF complex member Bromodomain-Containing Protein 9 (BRD9) was identified as a critical functional dependency in synovial sarcoma^432^ and is actively pursued for therapeutic intervention, including with degraders (Table 3). Importantly, the authors describe that mere inhibition of the BRD9 function only targets a single functionality of the target protein while leaving its scaffolding role within the BAF complex unaffected and is thus insufficient to impair its oncogenicity. In contrast, deletion or degradation of BRD9 (or other members within multi-protein complexes) may present a more efficacious approach to impair all functions of a target protein.^432^

Taken together, there has been growing interest in developing TPD-based therapies for pediatric cancer since the majority is driven by oncoproteins that are TFs or proteins with scaffolding function which are considered “undruggable” by conventional occupancy-based small molecules.^413,414^ Two teams funded in 2024 by cancer grand challenges are working on the development of degraders for “undruggable” oncogenic drivers of childhood solid tumors (PROTECT, https://www.cancergrandchallenges.org/protect; KOODAC, https://www.cancergrandchallenges.org/koodac). In the next section, innovative PROTAC-based strategies to target TFs are discussed and provide examples of how key drivers in pediatric cancers that are out of reach for conventional drug design could be targeted in the future.

## 6 PROTACs Targeting Transcription Factors

PROTACs have the potential to become best-in-class medicines due to degradation of the target instead of inhibiting it, thereby resulting in a more complete blockage of the protein and an improved therapeutic window.^199,433^ However, the true promise of PROTAC lies within targeting proteins that were previously considered intractable or “undruggable” by conventional therapies.^3^ Traditional small molecules follow occupancy-driven pharmacology where high affinity inhibitors are developed to target the active or allosteric site on their target to impair the function of the POI, an approach that is not feasible for all targets.^3,434^ Instead, PROTACs use event-driven pharmacology by degrading the POI without the need for an active site, thereby expanding the druggable genome to particular targets that harbor resistance mutations, are protein aggregates, have isoform expression or various localization, gene amplification or protein overexpression, scaffolding function, subunits of larger functional complexes, or are considered “undruggable” such as TFs.^3,225,252,435–439^

Although PROTAC targets do not need an enzymatic site, a small-molecule binding site with moderate affinity is required that needs to be approachable by an E3 ligase.^3^ Schneider et al. examined publicly available data to characterize the “PROTACtable” genome to identify targets that would be most amenable to PROTACs and concluded that over 1000 proteins that have not been targeted by PROTACs could be good targets to pursue with PROTACs.^252^ This work was the first comprehensive assessment aimed at guiding future PROTAC target selection and development. In the following section, this review focuses on recent advances to target the TFs with PROTACs.

TFs control gene expression by either repressing or promoting transcription by binding to short DNA sequences and TF binding motifs, which are usually localized in the enhancer or promoter regions of the gene.^440–443^ Of the approximately 1600 TFs encoded in the human genome, many have been associated with different disease phenotypes.^442^ Hence, TFs play crucial roles in the development of diseases such as cancer and present important therapeutic targets.^444,445^ Only a few examples exist, where small molecule inhibitors were able to target TFs such as the hormone receptors AR^446–448^ and ER,^449^ as well as NF-κB,^450,451^ STAT3/5/6,^452–455^ MYC, and EWS::FLI1.^211,456,457^ Although major efforts have been made to target various transcription factors, TFs beyond nuclear receptors have been historically viewed as “undruggable” due to their structural disorder and the absence of defined small molecule binding sites.^458^ Considering the limitations of conventional small molecules in targeting TFs, this presents a unique opportunity for PROTACs, as they do not require high affinity binding to their target.^3^

Successful design of PROTACs against AR and ER has been discussed above, but there has been progress to target other TFs as well. Liu et al. leveraged the unique TF binding motifs to design TF-PROTACs (Figure 4A), a platform to degrade the TFs of interest by introducing DNA oligomers and harboring the TF binding motif into VHL-based PROTACs via click chemistry.^459^ Similarly, oligonucleotide-based PROTAC (O’PROTACs) utilized double-stranded oligonucleotides that are recognized by TFs to achieve E3-mediated proteasomal degradation of the target TF and were demonstrated to work effectively against two cancer targets lymphoid enhancer-binding factor 1 and ETS-related gene.^460^ A similar strategy was employed to target RNA-binding proteins utilizing small RNA mimics incorporated into so-called RNA-PROTACs.^461^ Moreover, c-MYC was shown to be successfully targeted by PROTACs using a threose nucleic acid-DNA bivalent binder (Figure 4B), which showed efficacy in vitro and in vivo in triple negative breast cancer models,^462^ as well as a multifunctional aptamer-based PROTAC, which was combined with the artificial cyclization and anti-PD-L1 aptamer-based delivery system to improve stability and delivery, showing antitumor effects in vivo.^463,464^ In addition, TF targeting chimera (TRAFTAC) was developed on the basis of HaloPROTACs (Figure 4C), where a double-stranded DNA/CRISPR-RNA chimera forms a ternary complex with the target TF and a dCas9-HaloTag7 fusion protein to degrade the TF of interest.^465^ Building on the first generation TRAFTACs, Samarasinghe et al. have developed a second generation TRAFTAC named oligoTRAFTACs, which contain an oligonucleotide that can be recognized by the TF and a ligand recruiting an E3 ligase,^466^ thereby bypassing the requirement of Halo-tagged fusion proteins. The authors demonstrated successful degradation of c-MYC and brachyury using oligoTRAFTACs in vitro and in vivo in zebrafish. While these approaches resulted in effective degradation of the target TF, they rely on nucleic acids requiring transfection, microinjection, or additional cell-penetrating moieties to enter cells, which is a limitation of this technique from a translational perspective.^348,459,465–467^

**Figure 4:**
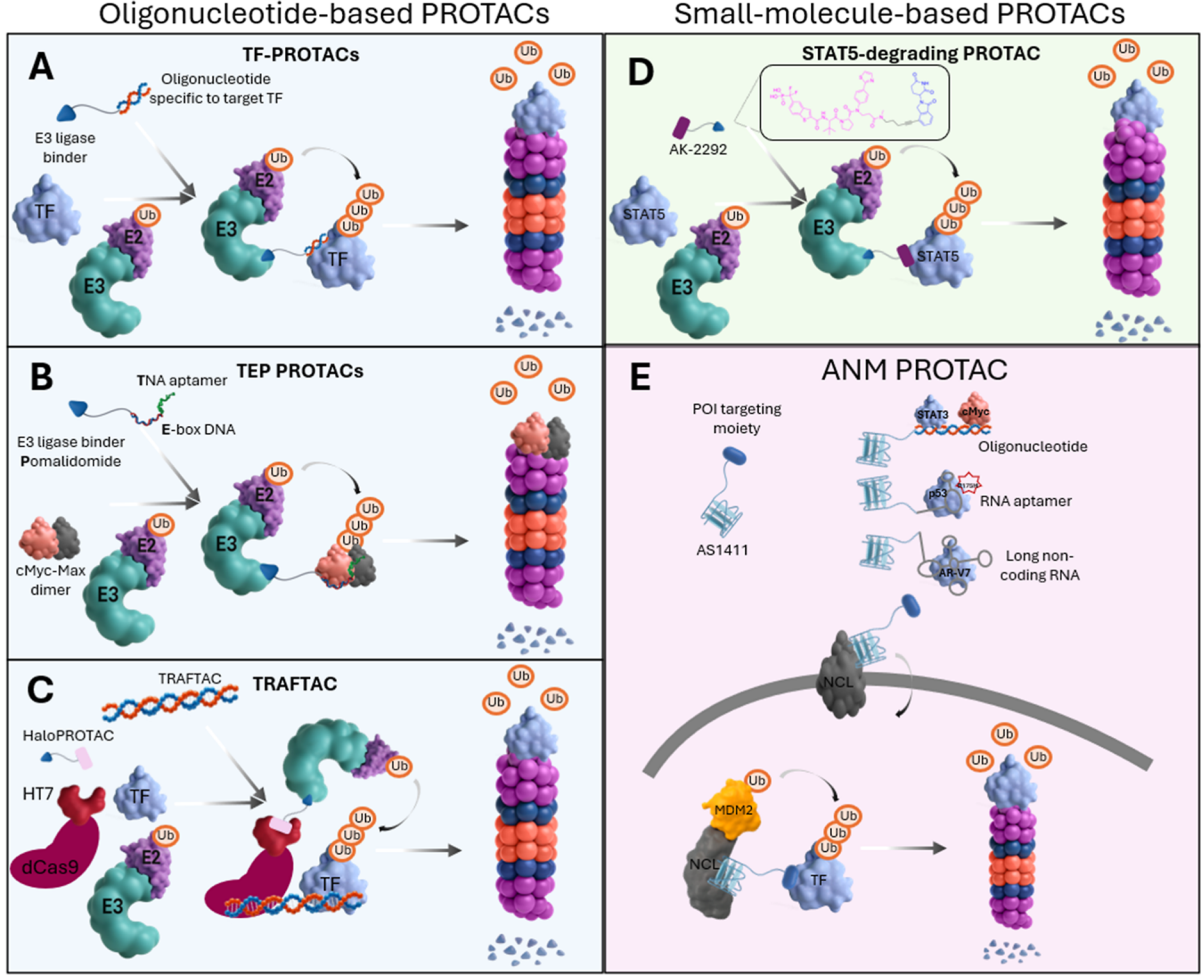
Different PROTAC strategies to target TFs. (A–C) Oligonucleotide-based PROTACs that use oligonucleotide sequences, which are selectively recognized by the TF of interest, coupled to an E3 ligase moiety to degrade the TF of interest. (D) Small-molecule-based PROTACs utilize existing small-molecule inhibitors of TFs to design PROTACs that are outperforming these small-molecule-inhibitors and show improved oral bioavailability over oligonucleotide-based PROTACs. (E) ANM-PROTACs contain AS1411 that recruits E3 ligase MDM2 via NCL and can be coupled to various TF-recruiting moieties that ultimately result in proteasomal degradation of the TF of interest. Through their NCL-targeting mechanism, AS1411-based ANM-PROTACs obtain tumor-targeting and cell-penetrating properties.

Moving beyond oligonucleotide-based PROTACs, Luo et al. used in silico modeling of the FOXM1 DNA-binding domain with existing small-molecule inhibitors, whose clinical application has been challenging, to design CRBN-based PROTACs. These PROTACs successfully degraded FOXM1 and demonstrated anti-tumor activity in vivo.^468^ In a similar fashion, Bai and colleagues developed the PROTAC SD-36, which selectively targets the TF STAT3, an attractive therapeutic target in cancer, by employing a STAT3 SH2 binder and demonstrated tumor regression in vivo.^437^ Interestingly, a PROTAC XD2-149 based on a STAT3 inhibitor napabucasin turned out to inhibit STAT3 signaling in pancreatic cancer cell lines; however, without proteasomal degradation of STAT3.^469^ Instead, XD2-149 degrades the oncogenic E3 ligase ZFP91 and reveals novel mechanisms of action of napabucasin. In addition to SD-36, AK-2292 was developed as small molecule PROTAC selectively targeting STAT5 (Figure 4D) showing efficacy in vitro and in vivo.^470^ Another example of TF-targeting PROTACs is reported by Yang et al., where SMAD3 was targeted by a VHL-based PROTAC attached to a SMAD3 binding moiety.^471^ In addition, Kymera Therapeutics is currently investigating PROTACs targeting STAT3 and STAT6 in clinical trials (Table 3).

These examples are small-molecule-based PROTACs with improved oral bioavailability and low toxicity that were more efficacious than the existing inhibitors targeting the respective TF.^472^ Together with the PROTACs targeting nuclear hormone receptors, they highlight the feasibility of PROTACs targeting TFs. However, these early examples of small-molecule-based and TF-targeting PROTACs had the opportunity to design PROTACs from existing small molecule binders to the TF, which is not the case for the vast majority of TFs. Thus, novel TF binders and innovative strategies must be discovered to enable targeting of TFs for which no alternative inhibitors are available.

One such approach may be using the recently described ANM-PROTACs. Fu et al. repurposed AS1411, a ligand to MDM2, conjugated to different TF recruiters, including DNA oligonucleotides targeting STAT3 and c-MYC, RNA aptamer for p53 mutant R175H, or a ligand derived from long non-coding RNA SLNCR1 to target AR splice variant 7 to generate ANM-PROTACs.^473^ In contrast to other discussed oligonucleotide-based PROTACs that have poor cell permeability without transfection reagents, ANM-PROTACs circumvented that issue. By recognizing a membrane-nucleus shuttling protein nucleolin (NCL) and anchoring the NCL-MDM2 complex via AS1411, penetration of these ANM-PROTACs into tumor cells was demonstrated (Figure 4E) as well as in vivo anti-tumor effects achieved via the degradation of the target TF in the absence of systemic toxicity.^473^ AS1411 is a nucleic acid aptamer^474^ designed to bind to NCL, which is often overexpressed in cancer cells,^475^ where it shuttles between the nucleus, cytoplasm, and cell surface, on which it acts as a membrane-anchored receptor.^476,477^ Although clinical results from AS1411’s use as an anti-cancer treatment showed insufficient response rates and suboptimal pharmacokinetics, its use was safe and well tolerated.^478–481^ Importantly, NCL interacts with the E3 ligase MDM2,^482^ which enabled the AS1411-induced recruitment of MDM2 through NCL, thus inducing proteasomal degradation of target proteins when AS1411 was incorporated in their PROTACs. Overall, the authors could take advantage of AS1411’s tumor targeting and cell-penetrating properties to design ANM-PROTACs that target “undruggable” targets.^473,483^ One limitation of this technique is the instability of AS1411 in the gastrointestinal tract, which necessitates its administration as an injectable medication.^473^

Taken together, there is a growing list of strategies to target TFs via innovative PROTAC designs. These initial efforts highlight the feasibility of therapeutically targeting disease-associated TFs and PROTAC’s potential as a new therapeutic modality.

## 7 Conclusion

Initially described in 2001,^6^ PROTACs have since enabled scientific discoveries and revolutionized the drug discovery field.^3,5^ Via the unique event-driven mechanism of action, PROTACs may surpass many conventional therapeutic strategies and have the potential to target proteins that were out of reach with the established drug design.^2–5^ Multiple PROTACs have entered clinical trials, and for one PROTAC, ARV-471, an NDA was recently submitted to the FDA.^367,368^ Inspired by the initial clinical success of PROTACs targeting well-characterized targets, the field is now moving toward targeting proteins that were previously considered “undruggable”^3^ with several innovative approaches demonstrating the feasibility of PROTACs targeting TFs. Oligonucleotide-based PROTACs have been successfully designed to target a variety of TFs, but may be limited in their clinical application due to poor cell permeability.^459,460,462,464–466^ Utilizing small-molecule inhibitors as POI-recruiter within PROTACs to target TFs improves oral bioavailability, but only a few small-molecule binders currently exist that target TFs.^437,468,470,471^ Given the rapid advancement of the TPD field over the last two decades, there is no doubt that there will be numerous novel innovative approaches in the near future designed to target the “undruggable” proteome as well as scientific discoveries enabled by TPD-based technologies.

## Abbreviations

AbTAC: Antibody-based PROTAC
AID: Auxin-induced degron
AR: Androgen receptor
ATTEC: Autophagosome tethering compound
AUTAC: Autophagy-targeting chimera
AUTOTAC: AUTOphagy-TArgeting Chimera
BRD9: Bromodomain-Containing Protein 9
BTK: Bruton tyrosine kinase
cIAP: Cell Inhibitor of Apoptosis Protein
CLIPTACs: Click-formed proteolysis targeting chimeras
CMA: Chaperone-mediated autophagy
CRBN: Cereblon
CsA: Cyclosporin A
DUBs: Deubiquitinating enzymes
ERG: ETS-related gene
FKBP: FK506- and rapamycin-binding protein
FRB: FKBP12-rapamycin-mTOR
GSPT1: G1 to S phase transition 1
HDACs: Histone deacetylases
HECT: Homologous to the E6AP carboxyl terminus
HIF: Hypoxia-inducible factor
IBR: In-between-RINGs
IPP: IκBα phosphopeptide
LEF1: Lymphoid enhancer-binding factor 1
LYTACs: Lysosome-targeting chimaeras
mCRPC: Metastatic Castration-resistant Prostate Cancer
MDM2: Murine double minute 2
NDA: New Drug Application
NCL: Nucleolin
O’PROTACs: Oligonucleotide-based PROTAC
PHOTACs: PHOtochemically Targeting Chimeras
POI: Proteins of interest
PROTACs: Proteolysis Targeting Chimeras
RING: Really Interesting New Gene
RBR: RING-IBR-RING
SARM: Nonsteroidal androgen receptor ligand
SERDs: Selective estrogen downregulators
SMASh: Small molecule-assisted shutoff
SNIPERs: Specific and non-genetic Inhibitor of Apoptosis Protein (IAP)-dependent protein erasers
TPD: Targeted protein degradation
TRAFTAC: Transcription factor targeting chimera
TFs: Transcription factors
UPS: Ubiquitin-proteasome system
VHL: Von Hippel-Lindau
WT: Wild type
